# Adult-born neurons add flexibility to hippocampal memories

**DOI:** 10.3389/fnins.2023.1128623

**Published:** 2023-02-15

**Authors:** Orsolya Fölsz, Stéphanie Trouche, Vincent Croset

**Affiliations:** ^1^Department of Biosciences, Durham University, Durham, United Kingdom; ^2^MSc in Neuroscience Programme, University of Oxford, Oxford, United Kingdom; ^3^Institute of Functional Genomics, University of Montpellier, CNRS, INSERM, Montpellier, France

**Keywords:** neurogenesis, memory, hippocampus, forgetting, pattern separation, flexibility

## Abstract

Although most neurons are generated embryonically, neurogenesis is maintained at low rates in specific brain areas throughout adulthood, including the dentate gyrus of the mammalian hippocampus. Episodic-like memories encoded in the hippocampus require the dentate gyrus to decorrelate similar experiences by generating distinct neuronal representations from overlapping inputs (pattern separation). Adult-born neurons integrating into the dentate gyrus circuit compete with resident mature cells for neuronal inputs and outputs, and recruit inhibitory circuits to limit hippocampal activity. They display transient hyperexcitability and hyperplasticity during maturation, making them more likely to be recruited by any given experience. Behavioral evidence suggests that adult-born neurons support pattern separation in the rodent dentate gyrus during encoding, and they have been proposed to provide a temporal stamp to memories encoded in close succession. The constant addition of neurons gradually degrades old connections, promoting generalization and ultimately forgetting of remote memories in the hippocampus. This makes space for new memories, preventing saturation and interference. Overall, a small population of adult-born neurons appears to make a unique contribution to hippocampal information encoding and removal. Although several inconsistencies regarding the functional relevance of neurogenesis remain, in this review we argue that immature neurons confer a unique form of transience on the dentate gyrus that complements synaptic plasticity to help animals flexibly adapt to changing environments.

## Introduction

Brain plasticity enables animals to encode novel information and adapt to changing environments. A leading hypothesis suggests that memory representations are stored within connected neuronal ensembles called engrams in each brain region and throughout the brain (Semon, 1921). Neuronal ensembles activated together by a learning experience undergo persistent functional modifications upon learning and are reactivated together during memory recall. Learning triggers lasting changes in synaptic strength between co-activated neurons (Hebb, 2005), often underlain by long-term potentiation (LTP) of relevant synapses (Bliss and Lømo, 1973).

**FIGURE 1:**
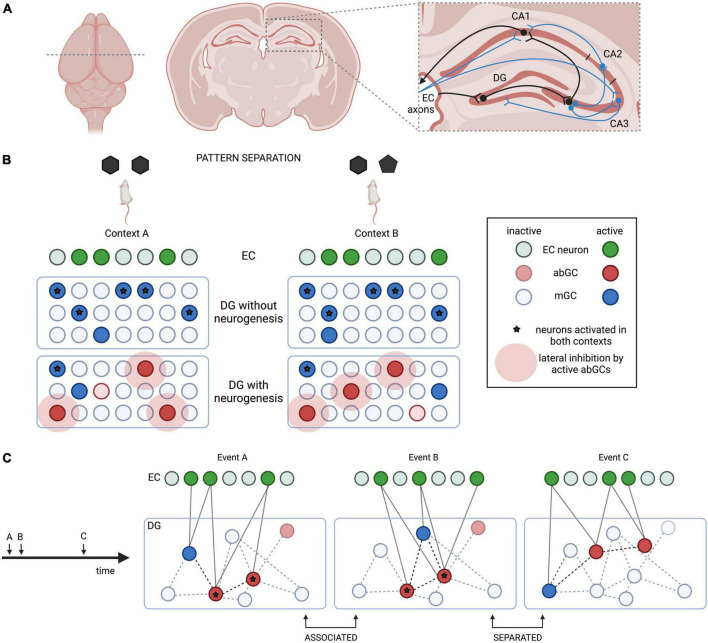
Simplified hippocampal circuitry and the role of adult neurogenesis in pattern separation in the dentate gyrus (DG). **(A)** Schematic hippocampal circuitry from a mouse brain coronal section. Beyond the basic trisynaptic loop (EC-DG-CA3-CA1, black), some entorhinal cortex (EC) inputs go directly to CA1/CA3, while some CA3 axons project to CA2, send collaterals to other CA3 neurons, and feed back to the DG (blue). **(B)** Adult-born GCs (abGCs) achieve pattern separation by limiting the activation of the same mature GCs (mGCs) in similar contexts. **(C)** “Time-stamping” could link contemporary events A and B, but separate remote event C by activating a changing population of abGCs.

In the mammalian brain, episodic-like memories are stored in the hippocampus (Figure 1A). The dentate gyrus (DG) of the hippocampus integrates spatio-temporal and event-specific information from the medial and lateral entorhinal cortex (EC), respectively, and converts them into sparse neuronal representations (Engin et al., 2015). Pattern separation enables highly analogous memories to be stored with little interference in distinct cell ensembles in the CA3 subfield (Leutgeb et al., 2007). The DG also attenuates the generalization of remote fear memories and may be involved in the remote memory retrieval (Bernier et al., 2017). CA3 performs the complementary process of pattern completion, enabling behavioral expression of a memory trace, even when the context or inputs of memory recall are different from encoding or incomplete (Leutgeb et al., 2007). Outputs from CA3 are compared with direct EC inputs in CA1 and sent back to the EC to be distributed across the neocortex for long-term storage. The EC also has direct connections with CA3, which are involved in discrimination of distinct stimuli (Fyhn et al., 2007).

In most mammals, hippocampal circuitry is constantly reformed by a unique form of structural and functional plasticity involving neurogenesis. Adult neurogenesis in rodents is present in at least two areas: the subventricular zone lining the lateral ventricles, and the subgranular zone of the DG (Lois and Alvarez-Buylla, 1993; Kempermann et al., 1997a). The former gives rise to cells that migrate to the olfactory bulb and differentiate into inhibitory olfactory neurons, while the latter generates excitatory glutamatergic granule cells (GCs). The vast majority of DG GCs are born perinatally, after which neurogenesis declines and is maintained at varying levels throughout adulthood (Ngwenya et al., 2015; Hochgerner et al., 2018). Of all DG GCs, ∼0.2% are generated daily in rats and ∼0.06% in mice (Kempermann et al., 1997a; Cameron and Mckay, 2001). Around half of adult-born GCs (abGCs) generated are eliminated through waves of programmed cell death during their maturation (Dayer et al., 2003; Ryu et al., 2016; Pilz et al., 2018). Surviving cells are stably maintained and ultimately become indistinguishable from developmental GCs (Dayer et al., 2003; Kempermann et al., 2003). Neurogenesis is balanced by the continuous removal of mostly perinatally-generated mature GCs (mGCs) (Ciric et al., 2019), resulting in a constant or slightly expanding DG cell number (Rapp and Gallagher, 1996; Kempermann et al., 1997b). In this review, we argue that integration of abGCs into the DG network confers plasticity to the classical cortico-hippocampal circuit.

## Functional integration of adult-born neurons

Proliferation of neural progenitor cells (NPCs) in the subgranular zone of the DG produces neuronal fate-committed cells that undergo stereotypic stages of maturation (Hochgerner et al., 2018; Pilz et al., 2018). Maturing abGCs extend dendrites and an axon toward CA3 (or CA2; Llorens-Martín et al., 2015) of the hippocampus (Zhao et al., 2006), shifting excitation-inhibition balance. The ensuing critical period of hyperexcitability (Mongiat et al., 2009; Danielson et al., 2016; Li L. et al., 2017) is characterized by lower LTP induction threshold and higher LTP amplitude compared to mature mGCs (Schmidt-Hieber et al., 2004; Ge et al., 2007; Li et al., 2013). Maturing abGCs continuously reform their connections with the local circuitry, in an activity-dependent manner (Toni et al., 2007; Jungenitz et al., 2018).

During early maturation, abGCs receive inhibitory inputs from local interneurons and form transient direct connections with mGCs (Hendricks et al., 2017; Gozel and Gerstner, 2021). Electrophysiological recordings show that abGCs receiving lateral EC inputs inhibit mGCs, while abGCs receiving medial EC inputs excite mGCs (Luna et al., 2019). Later, abGCs switch from direct interactions to synaptic competition with mGCs for EC inputs and CA3 targets. Electron microscopy evidence shows that abGCs initially contact pre-existing axon terminals occupied by other neurons, but later outcompete mGC axons to become unique synaptic partners (Toni et al., 2007). Similar processes occur dendritically (Toni et al., 2008; McAvoy et al., 2016). Firing connections are stably maintained while inactive ones are pruned, therefore hyperexcitable abGCs tend to prevail, driving the elimination of existing mGC connections (Tashiro et al., 2007; Yasuda et al., 2011; Restivo et al., 2015; Murray et al., 2020). The selective survival of abGCs may also be regulated by synaptic activity, and in an information-specific manner (Tashiro et al., 2006). abGCs additionally form dynamic connections with hippocampal interneurons that inhibit neighboring mGCs (lateral inhibition), and exert inhibition even on CA3 and CA1 (feedforward inhibition) (Chawla et al., 2005; Guo et al., 2018; Berdugo-Vega et al., 2020). This results in the overall sparsification of population firing across the hippocampus (Lacefield et al., 2012; Temprana et al., 2015; McHugh et al., 2022).

Excitation or artificial LTP induction, as well as exposure to new experiences, such as spatial learning, voluntary exercise, or increased sensory stimulation (e.g., animal housing in groups, novel toys in home cages etc.) promote neurogenesis (Kempermann et al., 1997b; Gould et al., 1999; van Praag et al., 1999; Deisseroth et al., 2004; Bruel-Jungerman et al., 2006; Darcy et al., 2014). Stress, aging, and some neuropsychiatric conditions decrease proliferation rates and responsiveness of abGCs (Ben Abdallah et al., 2010; Snyder et al., 2011). This may contribute to intensified stress responses (Snyder et al., 2011), and decreased learning abilities in older animals (Montaron et al., 2020).

## Contribution of adult-born neurons to memory encoding

The DG converts EC inputs into highly decorrelated representations in CA3 (Fyhn et al., 2007). This is achieved by changes in the correlated activity of the same sparse subset of GCs between similar contexts, such as when rats explore enclosures of slightly different shapes. Niibori et al. (2012) measured the overlap between CA3 cells activated during encoding and re-exposure using a cellular imaging approach, and found that suppressing neurogenesis disrupts the decorrelation of highly overlapping (but not dissimilar) contexts. Behavioral evidence suggests that critical period abGCs are important for tasks requiring separation of highly similar contexts, such as contextual fear discrimination or re-learning of a shock zone location (Table 1); however, these cells appear dispensable for learning a location in the water maze (Sahay et al., 2011, Burghardt et al., 2012). Intriguingly, blocking all outputs from GCs older than 3–4 weeks improves contextual fear conditioning (CFC) performance, suggesting that pattern separation not only relies on abGCs, but could be counteracted by mGCs (Nakashiba et al., 2012). abGCs might recruit inhibitory circuits to limit the activation of the same GCs in similar contexts *via* lateral and feedback inhibition, providing a potential mechanism for pattern separation (Kitamura et al., 2009; Engin et al., 2015; Temprana et al., 2015; Figure 1B).

**TABLE 1:** Key studies on the effect of hippocampal neurogenesis on behavioral pattern separation and forgetting.

Experiment	Neurogenesis manipulation	Manipulation approach	abGC identification	Performance	Subjects (sex, age)	Supports involvement of abGCs?	References
**Pattern separation**							
CFC	↑	Genetic Bax ablation in NPCs	Dcx, BrdU	↑	MF 14–18 weeks	✓	Sahay et al., 2011; Besnard and Sahay, 2021
	↓	X-ray irradiation	Dcx	↓		✓	
	↓	Nestin-rtTA/Tet mice	CldU	↓	M 8 weeks	✓	Tronel et al., 2010
	Ablation	Nestin-HSV-TK mice	Ki67, NeuroD	↓ When contexts similar	M 10 weeks	✓	Niibori et al., 2012
Touchscreen location discrimination	↑	Voluntary exercise	BrdU	↑	M 3–22 months	✓	Creer et al., 2010
Radial arm maze Touchscreen location discrimination	↓	X-ray irradiation and viral Wnt knockdown	Dcx	↓ When contexts similar	F 8+ weeks	✓	Clelland et al., 2009
CFC with changed shock zone	Ablation	X-ray irradiation of GFAP-TK mice	Dcx	↓	M 10+ weeks	✓	Burghardt et al., 2012
Water maze	Ablation	Genetic Bax overexpression in NPCs	BrdU, Dcx, apoptotic marker	↓	M 14 weeks	✓	Dupret et al., 2008
Novel object recognition	↑	Voluntary exercise	Dcx	↑ When contexts similar	F 8+ weeks	✓	Bolz et al., 2015
Continuous novel object recognition	Silencing 4–7 weeks old abGCs	Optogenetic silencing in abGC-ArchT mice	Opto-tagging	↑	M 4–6 months	✓	McHugh et al., 2022
CFC	Postnatal ablation	DNMT1 knockout	BrdU	↑ In M	MF 3–5 months	X	Cushman et al., 2012
Touchscreen location discrimination	↓	GFAP-TK mice	Dcx	↑ In reversal phase	M 8+ weeks	X	Swan et al., 2014
Water maze	↓	GFAP-TK rats	Dcx	No effect ↓ Under cold-water stress	*M 12+ weeks	X	O’Leary et al., 2021
**Neurogenesis-mediated forgetting**							
CFC Water maze Incidental context learning	↑	Voluntary exercise or proneurogenic drugs	Retrovirus-driven GFP, Dcx, Ki67	Increased forgetting	? 8+ weeks	✓	Akers et al., 2014
	↓	Post-training temozolomide treatment or TK^+^ mice		Improved retention		✓	
Water maze Odor-context paired-associates learning	↑	Voluntary exercise	Dcx	Increased forgetting but improved reversal learning	MF 8+ weeks	✓	Epp et al., 2016
	↓	Post-training vanganciclovir treatment or TK+ mice		Exercise failed to induce forgetting		✓	
CFC Water maze Paired associates learning	↑	Voluntary exercise	Dcx	Increased forgetting and improved reversal learning	*M ?	✓	Scott et al., 2021
CFC	↑	Voluntary exercise or p53 knockout	Dcx	Increased forgetting of recent memories	MF 8+ weeks	✓	Gao et al., 2018
CFC	↑	Memantine treatment	BrdU	Increased forgetting of remote memories after long re-exposures to training context	M 8+ weeks	✓	Ishikawa et al., 2016
Paired associates learning	↑	Voluntary exercise	Dcx	Increased forgetting	↑	✓	Epp et al., 2021
Water maze	↑	Voluntary exercise	BrdU, Dcx	No effect	*M 6+ weeks	X	Kodali et al., 2016
Water maze	↓	Post-training γ irradiation	BrdU, Dcx	No effect	*M 6+ weeks	X	Snyder et al., 2005

Information not reported in papers labeled with “?.” Most studied use mouse models, while those labeled *use rats. M, males; F, females. Dcx, doubleortin (1–3 weeks old neurons); BrdU, CldU: thymidine analogs (proliferating cells); Ki67 (proliferating cells); NeuroD (immature neurons).

Despite these advances, the extent of abGC contribution to pattern separation remains ambiguous, largely due to inconsistencies around defining and manipulating relevant neuronal populations, and in the behavioral paradigms used (Table 1). More specific DG-based pattern separation paradigms, optogenetic manipulations, and simultaneous recordings of abGCs and mature hippocampal cell types may help elucidate the precise role of abGCs in memory formation. Disrupted pattern separation in some, but improved performance in other hippocampus-based tasks suggests that abGCs may serve additional functions beyond pattern separation, depending on the behavioral paradigm.

Aimone et al. (2006) proposed that abGCs link memories encoded in close succession, while separating remote memories (Figure 1C). Because hyperexcitable DG cells are preferentially included into engrams (Park et al., 2016), critical period abGCs may be more readily recruited into any memory trace. This generates overlapping representations in CA3 that can be activated by the context represented in any of the temporally associated engrams. Therefore, integration of young abGCs into hippocampal engrams may help connect contemporary memories (Cai et al., 2016), while more mature abGC populations support pattern separation (Aimone et al., 2010). The involvement of juvenile-born GCs (Kesner et al., 2014) in “time-stamping” has been described in a task where animals use spatial cues to generate preference for a temporally paired spatial location. Lesions in either cell population eliminated preference for the cued location, suggesting disrupted associations between events occurring close in time.

Recently, new findings have questioned the validity of the “time-stamping” hypothesis. Whereas abGCs are indeed more likely to be recruited during spatial memory encoding and activated during retrieval (Kee et al., 2007; Trouche et al., 2009; Stone et al., 2011; Gu et al., 2012; Martinez-Canabal et al., 2013), little overlap was found between abGCs activated during encoding or retrieval of contextual fear memories (Kumar et al., 2020). This suggests that either abGCs are activated by behavioral states rather than specific events or contexts (Erwin et al., 2020), or that limited overlap between the two populations could be a general property of DG engrams (Denny et al., 2014). Another line of recent findings shows that maturing abGCs maintain their hyperexcitable properties for several months beyond the proposed critical period. These studies injected rats with various thymidine analogs to birthdate GC populations before quantifying their activity using immediate early gene expression. abGCs remained excitable especially in younger animals and animals that were offered environmental stimulation, and their activation supported learning even in older animals (Ohline et al., 2018; Montaron et al., 2020). This questions the idea of temporal integration and the long-standing view that abGCs exert their memory-related functions merely during their first weeks of existence (Alme et al., 2010).

## Adult-born neurons and memory consolidation

abGCs stably integrated into the hippocampal circuitry may also influence later stages of memory processing. Classical views of systems consolidation have held that after encoding, memories progressively lose their hippocampal dependence before transferring completely to neocortex (Frankland and Bontempi, 2005). Prefrontal cortex engrams are strengthened by CA3 and CA1 ripples (Nakashiba et al., 2009), while hippocampal engrams are gradually silenced (Kitamura et al., 2017; Gao et al., 2018).

Some evidence suggests that abGCs promote memory consolidation during sleep, when hippocampal and neocortical engrams are reactivated, and synapses are selectively strengthened or renormalized by dendritic remodeling (Mirescu et al., 2006; de Vivo et al., 2017; Li W. et al., 2017). abGCs active during CFC learning are reactivated during rapid eye movement (REM) sleep, and both optogenetic stimulation and silencing of reactivated abGCs disrupt consolidation (Kumar et al., 2020). Blocking neurogenesis reduces non-REM sleep and disrupts consolidation-related oscillations and cortex-hippocampus interaction, leading to poor spatial memory performance (Sippel et al., 2020). Further, the rate of neurogenesis also seems to determine the hippocampus-dependent period of memories (Kitamura et al., 2009). Although none of these studies directly links abGC engrams to these changes, or specifically accounts for sleep-induced changes in neurogenesis levels, they do demonstrate that abGCs are involved in sleep-related consolidation.

## Neurogenesis affects memory stability and causes forgetting

Hippocampal representations of consolidated remote memories are reactivated upon retrieval. This destabilizes engrams, allowing protein synthesis-dependent reconsolidation processes to update, strengthen, or silence them (Suzuki et al., 2004). Both immature and critical period abGCs are reactivated during retrieval, however, blocking protein synthesis in the immature population alone affects reconsolidation (Lods et al., 2021). Updating of memories is impaired in the novel object recognition task when an even younger abGC population is ablated, further supporting that highly immature abGCs mediate reconsolidation (Suárez-Pereira and Carrión, 2015). The emerging unique roles of abGCs in post-encoding memory strengthening was recently demonstrated; chemogenetic stimulation during retrieval of abGCs, but not mGCs, improved remote memory strength and accuracy in rats (Lods et al., 2022).

A growing body of evidence suggests that increased neurogenesis after memory encoding promotes forgetting (Table 1). For instance, pharmacologically enhancing neurogenesis increases the forgetting of remote CFC memories after long re-exposures to the original context make them return to the hippocampus (Ishikawa et al., 2016). This has been explained by gradual elimination of existing connections through synaptic competition with abGCs (Murray et al., 2020), and reduction in LTP persistence through feedback and feedforward inhibition (Alam et al., 2018). Neurogenesis also disrupts perineuronal nets in CA1, which otherwise protect memories from degradation by limiting interneuron activity (Evans et al., 2022).

Removal of a small number of connections may only reduce memory precision, allowing recall by cues slightly different from the original encoding context (generalization) (Ko and Frankland, 2021). This might involve pruning of synapses that mediate feedforward inhibition (Ruediger et al., 2011). Once more connections are weakened, memories become inaccessible. High postnatal neurogenesis may even explain why early childhood memories are forgotten in many species (infantile amnesia) (Akers et al., 2014). Memory representations are not fully erased, as optogenetic reactivation of DG engrams can partially recover these memories (Guskjolen et al., 2018).

Replacement of old memories with updated novel memories can occur in similar contexts without interference. Indeed, neurogenesis is involved specifically in tasks requiring high cognitive flexibility, such as re-learning of a changed spatial location. In this case, ablation of neurogenesis prevents, while expansion of the abGC population promotes better search strategies (Garthe et al., 2009; Burghardt et al., 2012; Swan et al., 2014; Berdugo-Vega et al., 2021). Increased post-training neurogenesis weakens memories acquired in the water maze, which ultimately enables later re-learning of the task (Epp et al., 2016). Therefore, abGCs might promote forgetting to subsequently support encoding of novel memories.

## Discussion

Neural circuits require flexibility to adapt to changing environments, and stability to preserve information. The brain uses two main approaches to achieve transience: synaptic plasticity and cellular plasticity, or neurogenesis. Turnover of dendritic spines is undoubtedly the primary mechanism of structural plasticity behind learning, raising the question of why the DG needs neurogenesis beyond the synaptic modulation of mGCs.

Memory encoding by abGCs adds an anterograde form of transience to the hippocampus. Computational models support that abGCs optimize the balance between pattern separation and completion (Becker, 2005; Weisz and Argibay, 2009; O’Donnell and Sejnowski, 2014; Finnegan and Becker, 2015). abGCs might be used specifically to incorporate information about new experiences into engrams in familiar contexts (Aimone et al., 2009). Importantly, the inherent temporality of neurogenesis could hardly be replicated by synaptic plasticity. Most research into temporal sequence generation in the hippocampus has focused on CA1 and CA2 (MacDonald et al., 2013; Mankin et al., 2015), but the contribution of DG abGCs merits further investigations.

abGCs also confer retrograde transience on DG engrams through weakening and elimination of existing connections. Novel DG engrams may be “overfitted” and thus require generalization for optimal expression through neurogenesis, which acts as a regularizer in neuronal networks (Richards and Frankland, 2017; Tran et al., 2022). By eliminating unnecessary details while maintaining core features, neurogenesis may make memories easier to recall in changing or noisy environments. Neurogenesis also helps “clear up” remnants of remote hippocampal engrams already consolidated in the cortex, similar to sleep that serves the same function on a shorter timescale (Alam et al., 2018). Models support that neurogenesis makes room for new memories and prevents interferences (Wiskott et al., 2006).

As brains have become more complex throughout evolution, neurogenesis in the DG was maintained and repurposed. Some argue that it confers key functional benefits that underpin the evolutionary success of mammals (Kempermann, 2012), while others dismiss it as an evolutionary remnant, given its low rates, especially in highly cognitively developed species. As neurogenesis is associated with energy costs, oxidative stress, and oncogenesis (Walton et al., 2012; Batista et al., 2014), its maintenance may only be beneficial in animals that need to flexibly adapt to rapidly changing or enriched environments (Abrous et al., 2021). Indeed, most generalists (e.g., rodents) show neurogenesis, but mammals living in stable or homogenous environments do not (e.g., cetaceans).

The outstanding cognitive abilities of the human brain are thought to result from plasticity. Yet, the maintenance of DG neurogenesis throughout adulthood remains debated (Boldrini et al., 2018; Sorrells et al., 2018), mainly due to a lack of non-invasive research methods. Single-nucleus RNA sequencing recently verified the presence of scarce immature GCs in the adult human DG, with a marked reduction in Alzheimer’s disease (Zhou et al., 2022). Whether these cells are actively generated during adulthood or retained in an immature state is unclear. Further research is required to establish if human neurogenesis has any cognitive benefits or functional implications in neuropsychiatric conditions (Mishra et al., 2022).

This review supports the idea that abGCs can participate in the formation of hippocampal memories and influence mGCs to help encoding, generalization, and forgetting. abGCs bring transience to the hippocampus both by adding and removing information about new events, experiences, or environments. Experimental standardization and technological advances can help resolve contradictions in the literature, for example, by combining abGC labeling, *in vivo* recording with engram cell- and synapse-tagging (Choi et al., 2018), and more advanced DG-specific behavioral paradigms. Standardized definitions of abGC versus mGC populations should also help draw clearer conclusions. Nevertheless if one accepts that, in addition to preserving information, a major goal of memory is to optimize behavior, a large body of evidence now supports adult neurogenesis as a meaningful contributor to hippocampal memory functions.

## Author contributions

This work was originally written by OF as part of a 3rd year literature review assignment at Durham University. OF and VC: conceptualization. OF, ST, and VC: writing. VC: supervision. All authors contributed to the article and approved the submitted version.
